# A multilevel longitudinal study of obsessive compulsive symptoms in adolescence: male gender and emotional stability as protective factors

**DOI:** 10.1186/s12991-017-0165-z

**Published:** 2017-11-22

**Authors:** Vasilis Stavropoulos, Kathleen A. Moore, Helen Lazaratou, Dimitris Dikaios, Rapson Gomez

**Affiliations:** 1 National and Kapodistrian University of Athens, Vas Sofias 72, 11528 Athens, Greece; 2 Federation University Australia, Mount Helen, Ballarat, VIC Australia

**Keywords:** Obsessive compulsive symptoms, Adolescence, Gender, Development, Emotional stability

## Abstract

The severity of obsessive compulsive symptoms (OCS) is suggested to be normally distributed in the general population, and they appear to have an impact on a range of aspects of adolescent development. Importantly, there are individual differences regarding susceptibility to OCS. In the present repeated measures study, OCS were studied in relation to gender and emotional stability (as a personality trait) using a normative sample of 515 adolescents at ages 16 and 18 years. OCS were assessed with the relevant subscale of the SCL-90-R and emotional stability with the Five Factor Questionnaire. A three-level hierarchical linear model was calculated to longitudinally assess the over time variations of OCS and their over time links to gender and emotional stability, while controlling for random effects due to the nesting of the data. Experiencing OCS increased with age (between 16 and 18 years). Additionally, male gender and higher emotional stability were associated with lower OCS at 16 years and these remained stable over time. Results indicate age-related and between individual differences on reported OCS that need to be considered for prevention and intervention planning.

## Background

Over the past two decades, significant emphasis has been placed on understanding the etiology of obsessive compulsive symptoms (OCS) [1, 2]. OCS entail recurrent and persistent thoughts that are experienced as intrusive, but which cannot be ignored (obsessions). Individuals often engage in repetitive physical or mental acts (compulsions) aimed at reducing or removing the stress induced by the obsessions. The severity of OCS is suggested to be normally distributed in the general population and often constitutes a transient part of normal development (e.g., commonplace childhood rituals such as not walking on pavement lines) [3, 4]. However, OCS over a specific threshold may result in obsessive compulsive disorder (OCD), which is a chronic psychiatric condition, with potentially serious repercussions [5] OCD includes either obsessions or compulsions or a combination of both. It tends to compromise the quality of life and the well-being of the individual in significant ways by causing distress and interfering with everyday functioning [1, 3].

Research has advanced knowledge regarding the nature and the etiology of OCS [6]. In particular, OCS have been described as heterogeneous, varying across several different dimensions (i.e., cleaning/contamination, forbidden thoughts, symmetry/ordering-counting, hoarding/acquiring and retaining objects) [3, 7, 8]. The broader OCS dimensions (content of OCS) experienced by individuals remain relatively stable over time (i.e., propensity to experience forbidden thoughts is likely to shift from thoughts of violence to thoughts of religion, but is less likely to shift from forbidden thoughts to hoarding [1, 9]; however, the severity/intensity of OCS may vary over developmental phases [1]. For instance, obsessions related to fear and loss of others are typically higher in childhood and sexual obsessions tend to present more during adolescence [10]. Although there is consensus that levels of OCS fluctuate over developmental phases, there is a dearth of longitudinal studies that focus specifically on factors associated with particular developmental trajectories [1]. As it is considered a high-risk period for the onset of OCS and the diagnosis of OCD, explaining variations in the severity of OCS during adolescence appears particularly important [2]. Also, identifying factors that may contribute to higher OCS severity in adolescents could provide useful clinical guidelines for more effective prevention and treatment interventions.

### Conceptual framework

To address these needs an integrative, multilevel approach that blends elements and concepts from the OCS literature and from the risk and resilience framework was used [4, 11, 12]. Specifically, Abramowitz et al. [4] contended that OCS may often constitute a part of normal development that may be better approached dimensionally, that is on a continuum from minimum to maximum OCS, rather than categorically (presence vs absence of OCS). In that context, pathological aspects of OCS have been defined as extreme versions of normative cognitive and emotional processes [11]. There is evidence supporting a multidimensional model of OCD/OCS, where the complex clinical presentation of OCD has been summarized through a combination of a number of consistent, temporally stable symptom dimensions. These are conceptualized on a spectrum of likely coexisting syndromes that may embrace normal obsessive–compulsive phenomena extending beyond the traditional nosological boundaries of OCD [13]. Subsequently, from an evolutionary psychology perspective, it is assumed that obsessions and compulsions derive from a mental human module that unconsciously produces risk scenarios. Within this framework, obsessions act as unintentional and ego-dystonic (e.g., not aligning with the person’s ego driven choices) indirect-medium or longer term risk avoidance mechanisms, which lead to future risk avoidance behaviors (this function is different to anxiety which aims to decrease immediate and direct risks [14]. These approaches to OCS are in accord with the risk and resilience theoretical framework, in which behaviors are supported to constantly vary because of the interplay of developmental (age-related), individual and contextual risk and protective factors [11]. To better investigate the effects and the interactions of risk and protective factors across each of the levels involved (age-related changes, individual and contextual), the risk and resilience framework is best employed using multiple levels of analyses [15, 16]. Such models of analyses enable the investigation of lifespan variations across individuals controlling for ecological-contextual effects. Accordingly, the present longitudinal study examined OCS dimensionally using a normative sample of Greek adolescents (assessed at 16 and again at 18 years of age) to determine the effects of potential risk (i.e., over time changes) and protective factors (i.e., gender and emotional stability).

### Adolescence and OCS

In terms of age-related factors associated with variations in the severity of OCS, the focus in the present study was on adolescence, specifically the period between 16 and 18 years. Adolescence is a pivotal developmental stage [17]. It is a critical time for the development of OCS symptoms in general and the onset of OCD in particular [3, 18] and a time of multiple and concurrent life transitions including school, peer relationships, and family interactions [19]. These developmental turning points have been assumed to potentially trigger and/or exacerbate OCS among vulnerable individuals [6].

The period between 16 and 18 years is particularly critical for adolescents in Greece (from where the current sample was sourced). These years coincide with the first two grades of lyceum (secondary high school), during which period students become entitled to select, for the first time, the type of education they wish to pursue (academic or vocational track), as well as specific topics upon which their subsequent tertiary education entry exams are based [20]. Interestingly, this period of elevated educational accountability for Greek adolescents overlaps with a time of high prevalence of OCS [21]. Given that OCS have been suggested to have a gradual onset [3] and that prospective studies have contended that OCS severity varies over time [22] the need for longitudinal research to address this particular age range and population is compelling. In the present study, these potential age-related effects are studied in light of their interaction with gender and emotional stability.

### Gender

Gender has been repeatedly examined as a factor that differentiates OCS over the life course and during adolescence in particular [23–26]. Literature referring to OCD diagnosed patients contends that gender appears to affect OCS in regard to their expression (e.g., type of symptoms experienced across genders), their age of onset and their presented comorbid disorders [23]. Specifically, considering OCS types, while males appear to present more frequently sexual, religious, doubt and checking obsessions, and repeating compulsions, females seem to be more vulnerable to fears of contamination [23]. In regard to the age of initiation of OCS, males diagnosed with OCD incline to demonstrate an earlier onset than females, a more chronic pattern of symptoms and a greater social impairment [23, 24]. In that line, considering comorbidity, OCD male patients present more frequently with social phobia, tic and substance use disorders than their female counterparts, who tend to be higher in depression, suicidal thoughts, eating and impulse-control disorders [23, 24]. These findings are complimented by some adolescent community sample studies, which revealed a significantly higher OCS prevalence for males than females [27]. However, other findings based on clinical, as well as community samples, showed that gender did not associate with either the OCS heterogeneity or differences in the OCS prevalence rates [25, 28]. At this point, it should be noted that the international literature supports that females are at higher risk for anxiety and fear related symptoms, such as OCS are classified, partially due to socialization processes that encourage a feeling of sensitivity and vulnerability, which predisposes and precipitates anxious manifestations [26]. These inconsistencies considering gender’s effect on OCS and OCD between findings related to clinical and community samples, as well as studies across different age groups and national populations necessitate further examination.

### Emotional stability and OCS

Research has suggested several significant associations between individual level variables and susceptibility to OCS including different forms of psychopathological symptoms and impairments in executive functioning [29, 30]. In this context, OCS have been repeatedly related to personality traits [31–36]. The link between emotional stability, as a personality trait, and OCS is emphasized here. Emotional stability describes the level that a person presents to be emotionally stable under various conditions and not prone to anxiety, depression, and/or other types of high emotional fluctuations [37].

Inclusion of emotional stability in the multilevel conceptualization of OCS was prompted by several empirical findings and observations. High emotional stability (more frequently assessed by its antithesis, neuroticism) has been identified as an individual level resource for a range of psychopathological forms [38–40]. In particular, emotional stability (low neuroticism) has been repeatedly related to lower OCS and OCD in both community and clinical, adult and adolescent samples [32–34, 41–44]. While various instruments have been used to assess each construct, such as the several scales loosely entitled Big Five [45, 46], and Eysenck’s Personality Inventory [47] to assess personality traits and the Maudsley Obsessive Compulsive Inventory [48] Obsessive Compulsive Inventory [49] and the Y-BOCS [50] to assess OCD, results consistently demonstrate associations between the two.

Despite the significant body of research conducted in regard to the association between emotional stability and OCS, there is (to the best of the authors’ knowledge) a dearth of studies adopting a risk and resilience approach with an emphasis on the developmental period of late adolescence. Such an approach would require a longitudinal examination of the link between emotional stability and OCS, concurrently controlling (taking into consideration) for the effects of the proximal context/environment of the individual. Addressing this gap appears to be important as emotional stability presents age-related variations that seem to be more intense during late adolescence [51]. Specifically, emotional regulation skills, which have been closely associated with emotional stability [44, 52], have been shown to vary over adolescent developmental periods with mid adolescence showing the smallest repertoire of emotion regulation strategies [53]. Furthermore, social-investment theory suggests that personality maturation, which is interwoven with a gradual increase of emotional stability scores, is largely the outcome of normative life transitions to adult roles [51]. Subsequently, the period of late adolescence examined in the present study (16–18 years) is assumed to be characterized by progressively higher emotional stability scores that could have a progressively protective effect on adolescents’ vulnerability to OCS. This hypothesis is underscored by the fact that the age of onset of OCD is bimodal, with early-onset before 10 years and late-onset after the age of 17 [54]. Therefore, it can be argued that these two points in time truncate any possible correlation between age and OCS, which overlaps with a transitional period for the development of emotional stability. Interestingly, late adolescence, specifically 16–18 years of age is considered to involve high levels of change in personality traits, including emotional stability [55]; and is suggested to be a peak period for the onset of OCS [3, 18].

Similarly, emotional stability as a personality trait is expected to vary due to different contextual (i.e., classroom) effects [56]. It has been found that an individual’s behaviors and personality traits are calibrated by functionality requirements, which initiate as conditional adaptations that become more permanent the longer the person is exposed to the effects of a specific context [56, 57]. Such contextual effects (i.e., classroom) on levels of emotional stability could influence its association with OCS during late adolescence and, therefore, need to be controlled/addressed by the conducted analyses (i.e., in the present study random effects due to the classroom of the participants were controlled at level 3).

### The present study

This repeated measures research focuses on individual differences in OCS from 16 to 18 years in a normative sample of Greek adolescents. These differences were examined both between and within individuals, through the use of three-level hierarchical linear modeling (HLM) for analyzing nested data [20]. This process enables the investigation of intra-individual (over time changes within individuals) change along with between individual differences, controlling for random effects due to the nesting of the data (classroom of the individuals). The following research hypotheses were addressed:

#### H1

It is hypothesized that participants’ OCS scores will increase between the ages of 16 and 18 years. This is in accordance with previous studies that support an increase of OCS during developmental phases which are marked by critical life events, such as the nationwide entry exams sat by Greek adolescents hoping to gain entry to a tertiary institution [51].

#### H2

Based socialization processes that increase the vulnerability of females to OCS and gender related differences revealed in community sample adolescent samples, it is envisaged that male adolescents will report lower OCS scores both at the age of 16 and prospectively [26, 27].

#### H3

Based on the protective role of emotional stability for OCS and OCD, it is hypothesized that more emotionally stable adolescents will report lower OCS scores over time [32–34].

## Methods

### Participants

This survey received approval from: (i) The Ministry of Education, (ii) The Teachers’ Council, and (iii) Parents’ consent. The sample was selected from the Athens metropolitan area and a specific regional area using the method of randomized stratified selection based on the latest inventory card of the Ministry of Education (2010). The ratios of high schools and students were identified: (1) between the metropolitan area and the selected regional population and (2) between academic vs vocational track high schools. Based on these quotas participants were randomly (by lottery) selected at the classroom level (for exact quotas see Table 1).

**Table 1:** Original population and sample proportions

	Area of residence		Total
	Regional area (Korinthia)	Athens metro area	
Population			
Type of school			
Vocational track			
N	744	13,560	14,304
% of total population	.83%	15.12%	15.95%
Academic track			
N	2769	72,614	75,383
% of total population	3.09%	80.96%	84.05%
Total			
N	3513	86,174	89,687
% of total population	3.91%	96.08%	100%
Study sample			
Type of school			
Vocational track			
N	7	49	56
% of sample	1.40%	9.50%	10.90%
Academic track			
N	34	425	459
% of total sample	6.60%	82.50%	89.10%
Total			
N	41	474	515
% of total sample	8.00%	92.00%	100.00%

Population refers to the actual relevant student population of the Athens Metro Area and the Regional Area (Korinthia) in 2010 and sample to the study’s sample

The sample consisted of 515 Greek students embedded in 33 classrooms. *X* ^2^ analysis confirmed that the sample did not significantly differ from the original population regarding area of residence and the type of school of the participants *X* ^2^ = 1.58, DF 1,3, *p* = .66. Parents’ consent was 98% and the students’ response rate was over 95%. With respect to parents’ and guardians’ socioeconomic profile, 80.2% were married, 6.2% of the mothers and 6.8% of the fathers were unemployed, and 77.9% of the mothers and 64.5% of the fathers had completed education equal or above high school at time 1. The estimated maximum sampling error with a size of 515 is 4.32% (*Z* = 1.96, confidence level 95%).

Participants were assessed twice, two school years apart, and their responses matched with a unique code (Time 1: age *M* = 15.68 years, SD = .65, range 15.5–16.5, 53.6% females, 46.4% males; Time 2: age *M* = 17.67 years, SD = .54, range 16.5–17.5, 54.6% females, 45.4% males). The retention rate was high (72%) with attrition due to changes of school, and school and research drop outs. To evaluate the attrition effects, attrition was used as an independent variable (dummy coded 1 = Attrition, 0 = no attrition) at level 2 of the Hierarchical Linear Modeling-HLM analyses in order to assess whether it effected OCS scores and its associations with the other independent variables. Results confirmed that attrition did not have any significant effects on OCS symptoms and their association with emotional stability (Table 2).

**Table 2:** Assessment of the attrition effects in HLM analyses

	Fixed effects with Robust standard errors				
	*b*_*i*_	SE	*T*	DF	*P*_*i*_
Attrition	.07	.09	.76	32	.452
Emotional stability attrition*	.01	.13	.08	32	.934

Attrition refers to participants who did not complete two measurements. To evaluate the attrition effects attrition was used as an independent variable (dummy coded 1 = Attrition, 0 = not attrition) at level 2 of the HLM analyses to assess whether it effects OCS and their associations with emotional stability

### Instruments

#### Symptom Check List 90 Revised (SCL-90-R)

To assess OC, the relevant subscale of the SCL-90–R questionnaire [58] was used. This scale includes ten items and reflects symptoms typical of obsessive–compulsive disorder. The emphasis is on thoughts, impulses, and actions that are experienced as irresistible by the individual but are of an ego-dystonic or undesired nature (e.g., “Having to check and double check what you do?”, “Unwanted thoughts, words, or ideas that won’t leave your mind”, “Having thoughts about sex that bother you a lot”). Experiences of cognitive attenuation are also included in this dimension. Adolescents reported the intensity of their symptoms on a 5-point Likert scale (0 = *not at all* to 4 = *all the time*). Scores ranged from 0 to 4, where 0 indicated minimum and 4 indicated maximum symptoms. In the present study, the internal reliability of the OCS subscale was acceptable Cronbach *α* = .79. The use of the OCS subscale of the SCL-90 was preferred here due to its standardization for Greek samples and for reasons of comparability with other Greek studies [20, 59, 60].

#### Five Factor Questionnaire for Children (FFQ)

To assess emotional stability, the FFQ emotional stability subscale was used [61]. The questionnaire consists of five subscales: extraversion, emotional stability, conscientiousness, agreeableness, and openness to experience. Each subscale includes eight bipolar adjectives (e.g., “I am calm—I am hypersensitive”) that are answered on a 5-point scale (1 = very, 2 = somewhat, 3 = neither/nor, 4 = somewhat, 5 = very) situated in between. The mean for the emotional stability subscale was calculated resulting in a range from 1 to 5, indicating the minimum and the maximum presence of the trait. The internal consistency of the emotional stability subscale in the current was Cronbach *α* = .71.

### Procedures

The first time point assessments were collected in the school year 2009–2010 and the second time point assessments were collected in the school year 2011–2012. The process of data collection was identical between the two time points. A specially trained research team of 13 undergraduate, postgraduate, and PhD students of the Department of Psychology of the University of Athens collected the data in the participants’ classrooms during the first two or the last two school hours (45 min each) of a school day, according to the permission provided by the Ministry of Education. The adolescents were motivated to participate in the study by the fact that they would not have to attend subjects taught during the time of the study and they would not be considered as absent from lessons. It should be noted that according to the Greek school regulation, students are allowed to progress to the next grade on the condition that they have not exceeded 50 school hours of unjustified absence per school year.

### Statistical analyses

**Fig. 1:**
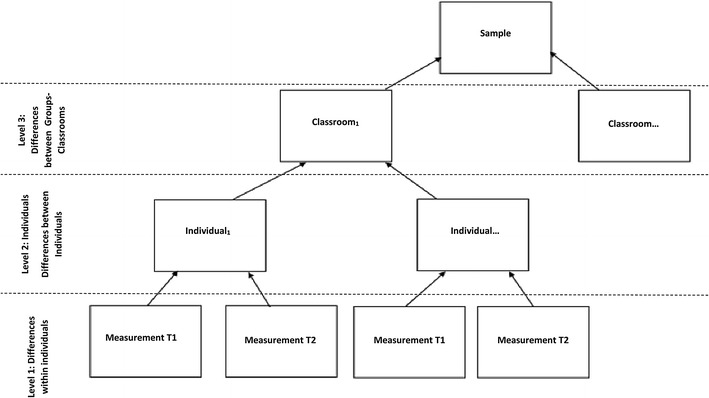
Multilevel Data Structure

Multilevel modeling was used to statistically analyze a data structure where measurements at two time points (Level 1) were nested within individuals (Level 2), who were nested within classrooms (Level 3) [1]. This approach was chosen to enable the study to disentangle and examine age-related changes on OCS at Level 1 and the effect of gender and emotional stability at Level 2, while controlling for possible random effects due to the nesting-clustering of the data (participants within classrooms) at Level 3 (see Fig. 1: multilevel data structure) [2]. Subsequently, HLM 6.0.8 software was used [62]. Model testing proceeded in successive phases, such that each of the examined conditions were first studied separately, before included in the full model (Raudenbush et al. [62]): (1) Unconstraint (null) model; (2) Random ancova model (Level 1 predictor); (3) Means as outcomes model (Level 2 predictors-gender and emotional stability); (4) Random coefficient (regression slope-full model) model (Levels 1 and 2 predictors-time and gender and emotional stability). Due to the results not being statistically significantly different, only the full model will be reported here. In this context, OCS (Level-1 outcome variable) were predicted for each individual at Level 1 by time in the study. Time was centered at time 1 such that the individual intercepts referred to the initial Level of OCS (Time 1 = 0, Time 2 = 1). The individual initial Level and the individual linear change over the two assessments (slope) were predicted at Level 2 by gender (females = 0, males = 1) and emotional stability. Finally, random effects due to the clustering of the participants were controlled through random effects equations at Level 3 in regard to both the main effects of time, gender, and emotional stability, as well as the cross-level interactions between time and emotional stability and time and gender (slopes). To control for mis-specification (i.e., lack of linearity) and the distributional assumptions at each level (lack of normality, heteroscedasticity), HLM results accounting for robust standard errors (which are insensitive to possible violations of these assumptions) were calculated. Considering missing values, whereas they do not present a problem at Level 1 in HLM and did not occur at Level 3 (classrooms), missing values at Level 2 (individuals) were addressed. Although they were unsystematic, to avoid listwise deletion, multiple imputation was applied (five Maximum Likelihood imputations using SPSS) using all available Level 2 variables. This type of imputation was selected as it outperforms listwise deletion for parameters involving many recouped cases and results to improve standard error estimates [63]. Based on previous literature, all multilevel analyses were calculated five times and their results were averaged [64]. Prior the HLM analyses, the means, standard deviations, inter-correlations between the HLM variables were estimated (Table 3).

**Table 3:** Means, standard deviations, correlations

Time	Mean	S D	1	2	3
1. Emotional stability time 1	3.11	.43			
2. Emotional stability time 2	3.30	.62	− .03		
3. OCS time 1	1.17	.70	− .10*	− .02	
4. OCS time 2	1.26	.69	.04	− .31*	.04

* *p* ≤ .05

## Results

To assure that the three levels contributed to variation in OCS scores, the level components were calculated from the unconditional model (*X* ^*2*^ = 1096.49, DF = 474, *p* = .001; *X* ^*2*^ = 44.25, DF = 32, *p* = .07). As an additional step, the intra class correlation (ICC) was calculated to determine which percentage of the variance in OCS is attributable to classroom membership (Level 3), which percentage is attributable to between individual differences (Level 2) and which to over time differences within individuals (Level 1). Results suggested that 42.32% (variance component = .204) of the variance in OCS is at the first Level (over time differences within individuals), 55.60% (variance component = .268) at Level 2 (the individual level) and 2.08% (variance component = .010) at Level 3 (between classrooms-controlled in the present analyses).

Therefore, HLM equations were calculated (see “Appendix”). The Level 1 intercept for the cross-sectional findings at the age of 16 years was 1.27 (this represents the estimated mean OCS score for adolescents of mean emotional stability controlling for random effects due to classroom participation). Considering how OCS change between 16 and 18 years (*hypothesis* 1), the time coefficient was *b* = .15 (*p* = .001). This indicated that the average OCS score increased to 1.42 (1.27 + .15 = 1.42) at the age of 18 for adolescents of mean emotional stability.

Gender was associated with OCS (*hypothesis* 2), *b* = − .20 (*p* = .012). Consequently, the average OCS score of male adolescents (0 = females, 1 = males) decreased to 1.00 (1.27 − .20) at the age of 16. Considering the effect of gender over time, the coefficient was *b* = − .11 (*p* = .144) indicating that the effect of the cross-level interaction of gender with time was not significant.

**Fig. 2:**
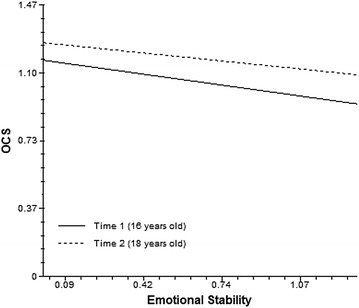
Emotional stability and OCS over time

Emotional stability was associated with OCS (*hypothesis* 3), *b* = − .19 (*p* = .001). Consequently, the average OCS score of adolescents who scored one point higher than the estimated mean in emotional stability decreased to 1.08 (1.27 − .19 = 1.08) at the age of 16. Considering the effect of emotional stability at time 1 on OCS at time 2, the coefficient was *b* = .49 (*p* = .300) indicating that the effect of the cross-level interaction of emotional stability with time was not significant (see slope on Fig. 2: Emotional stability and OCS over time). The complete model explained 11% of the overall OCS variance resulting from 5, 5, and 1% of Levels 1, 2, 3, respectively. Analyses controlled for random effects due to other individual level random effects and classroom nesting/clustering (see “Appendix” for equations) (Table 4).

**Table 4:** HLM analysis predicting adolescents’ OCS scores

	Presence score									
	Fixed effects without Robust standard errors					Fixed effects with Robust standard errors				
	*b*_*i*_	SE	*T*	DF	*p*_*1*_	*b*_*i*_	SE	*T*	DF	*p*_*1*_
Cross-sectional results										
Intercept	1.27	.04	29.83	32	.001	1.27	.04	32.30	32	.001
Emotional stability	− .19	.08	− 2.49	32	.004	− .19	.06	− 2.94	32	.001
Gender	− .20	.06	− 3.28	32	.003	− .20	.05	− 4.23	32	.006
Over time results										
Intercept (time)	.15	.05	3.12	32	.004	.15	.04	4.29	32	.001
Emotional stability	.06	.09	.69	32	.494	.06	.06	1.05	32	.300
Gender	− .11	.08	− 1.35	32	.187	− .11	.07	− 1.50	32	.144

Table 3 summarizes the main results regarding the individual factors examined and is divided into four parts. The upper left part presents the cross-sectional findings without controlling for random effects. The lower left part presents the over time change results without controlling for random effects. The upper right part presents the cross-sectional findings after controlling for random effects at Levels 2 (individual) and 3 (Classroom). The lower right part presents the over time change results after controlling for random effects at Levels 2 (individual) and 3 (Classroom). Controlling for random effects mildly differentiated the results, and therefore, only the results after controlling for random effects were considered and reported in the text (right side of the table)

## Discussion

In the present study, an integrative, multilevel approach that combined elements and concepts from the OCS literature and the risk and resilience framework was adopted to examine variations in OCS severity in a normative sample of Greek adolescents (assessed at 16 and 18 years of age). Specifically, the aim was to examine age-related change in OCS between 16 and 18 years taking into consideration the effects of emotional stability and male gender as individual level protective factors, while controlling for clustering (classroom) effects. This integrative framework was operationalized via a multilevel hierarchical linear model. The model was composed of three levels: the temporal factors (i.e., OCS over time), emotional stability, and gender as an individual level factors and controlled for random effects due to the nesting of the data (classrooms of participants). Multilevel analysis demonstrated that OCS increased between 16 and 18 years. Furthermore, male adolescents and adolescents higher in emotional stability were significantly less susceptible to OCS at the age of 16 years and these associations did not vary over time.

### Age-related changes and OCS

The effect of age on OCS severity has been studied repeatedly [1, 4, 10, 11] albeit with equivocal results. In our longitudinal assessment of a normative and representative sample of adolescents in Greece, the results revealed that OCS scores increased significantly between 16 and 18 years, encompassing one of the bimodal periods for onset of OCD. In another prospective study, Alvarenga et al. [5] reported a gradual increase of OCS in children aged 6–12 years which accords with the lower age peak for onset. However, in their study of adolescents diagnosed with OCD, De la Cruz et al. [1] found a tendency towards a negative relationship between OCD symptoms and age. These equivocal findings could be attributed to the different sampling methodologies (community vs clinical samples) or scales used and even the different age ranges and cultural backgrounds of the participants. However, the general consensus from past studies is that the increase of OCS and the onset of OCD occurs most often either during late adolescence, as found in the current study, or before the age of 10 [4, 18]. Indeed, Fineberg et al. [22] discussed the dearth of prospective studies which have considered the progression of OCS across developmental phases and recommended longitudinal research at these times with non-clinical populations.

The results of the present study support the results of past studies which have indicated that challenging life events exert a possible causal effect on OCS [4, 6]. In particular, Abramowitz et al. [4], Mataix-Cols et al. [65], and Stewart et al. [66] suggested that times of educational-family transitions and student exams may act as a trigger or exacerbate OCS among more vulnerable individuals. Such an event was certainly present for the current sample of students who were faced with their national university entrance examinations, which succeeded time point 2 measurement. It can be argued that students’ increased pressure associated with this educational challenge could contribute to the increase in the reported OCS scores in the current sample. Given that: (a) to the best of the authors’ knowledge this is the first prospective study of OCS severity in adolescents in Greece during the period between 16 and 18 years and; (b) the sample in the present study included exclusively high school students (and not late adolescents who were not attending lyceum/secondary high school), this conclusion needs to be addressed with caution.

Despite the need for more longitudinal and cross-cultural studies of typical adolescent development between 16 and 18 years, our results have direct implications for planning prevention and treatment initiatives. The need for more prevention resources and programs to be allocated to adolescents before the age of 16 years in order to prevent age-related OCS behaviors from escalating into clinical problems later in life is highlighted—especially for more vulnerable individuals.

### Gender

In terms of the individual level protective factors assessed here, being a male was found to significantly reduce OCS symptoms at the age of 16 and this effect remained stable over time. This finding, at least partially, agrees with past research illustrating that gender associates with differentiations in OCS (e.g., expression/type, age of onset, comorbidity; see above) [23]. However, the present finding appears inconsistent with regard to the direction of the differences in OCS across genders. Specifically, findings based on clinical samples have indicated that males tend to experience more intense OCS, interwoven with significantly higher social impairment [23, 24]. However, it should be noted that these differences refer to clinical samples, while the present study examined a normative community sample of Greek adolescents. Therefore, one could assume that while late adolescent males tend to present lower OCS levels in general, it is likely that when they present OCS to the extent that these might exceed the diagnostic thresholds, these tend to associate with higher levels of impairment than females. This hypothesis is consistent with differences in the phenomenology of other clinical manifestations across genders and between community and clinical samples [67, 68]. A potential explanation of this finding (e.g., adolescent males lower on OCS than females) could be viewed in the context of “gender appropriateness hypothesis” [68]. This suggests that females are more vulnerable to anxiety and fear related symptoms, including OCS, due to socialization effects that may cultivate a self-perception of sensitivity and vulnerability that attracts anxious manifestations [26]. Nevertheless, inconsistencies considering gender’s effect on OCS and OCD between clinical and community sample studies, various age groups and national populations are not uncommon and may indicate specific age, type of sample (clinical vs community), and national group limitations in the generalizability of the findings [23–26].

### Emotional stability and OCS

Emotional stability was additionally found to reduce the severity of OCS reported by adolescents. Other studies have similarly shown that higher emotional stability (lower neuroticism) is associated with decreased susceptibility to psychopathological symptoms in general [37, 39, 40] as well as OCS and OCD in particular [32–34, 41–43] by decreasing individuals’ vulnerability to pressure. In other fields of research, emotional stability has been associated with higher vulnerability to shame, psychological inflexibility, and emotional dysregulation [44] resulting in higher levels of anxiety. Lower emotional stability that increases anxiety could in turn trigger and/or reinforce OCS [8].

The current results suggest that the relationship between emotional stability and OCS does not significantly change in adolescents aged between 16 and 18 years old. This finding indicates that this association (and not emotional stability or OCS independently) is broadly stable during this specific developmental phase. In light of the significance of developmental timing, as highlighted in the risk and resilience framework [12], this finding may mean that adolescents low in emotional stability may have an earlier onset than late adolescence, and may be more vulnerable to developing OCS in subsequent times of stress. This is in line with international literature that has suggested such a gradual increase in the levels of emotional stability over developmental periods from early to late adolescence and emphasizes the need of emotional stability to be considered in the planning of OCS prevention and intervention initiatives [51]. In particular, OCS prevention and treatment programs in adolescence could include psychoeducation activities in regard to emotional stability (e.g., explaining how reacting calmly and stable to pressure may protect from the development of OCS symptoms); this would reinforce the individuals’ level of OCS risk awareness and self-reflection. However, given the paucity of relevant research, it is important to interpret this suggestion with caution.

### Conclusion, implications, limitations and further research

The present study illustrated the benefits of applying a longitudinal, contextualized methodology when investigating OCS in adolescence. This study’s strengths entail (a) the longitudinal design, (b) a normative and representative sample, and (c) the use of multilevel analyses that enabled the combined examination of developmental risks (i.e., aging between 16 and 18 years) and individual resources (i.e., emotional stability) in regard to OCS. Subsequently, the results have implications for OCS prevention and treatment in adolescence. Specifically, prevention or treatment strategies that would increase emotional stability levels are suggested to be more beneficial when applied before the age of 16 years in reducing OCS severity. Furthermore, Greek adolescents presenting OCS may benefit more by targeted group interventions that will aim to reduce the levels of pressure between 16 and 18 years [69]. Prevention initiatives and programs should ideally embrace an element of gender differentiation (during the period between 16 and 18 years) to address the progressively higher OCS risk in females, and include psychoeducation modules referring to teacher, parents, and mental health practitioners regarding the potential OCS developmental trajectory related increase between 16 and 18 years.

Despite its strengths, this study has limitations. First, findings were based on self-report questionnaires, while the use of the OC subscale of SCL-90 as the only measure of OCS restricted the investigation of OCS in a multidimensional fashion. Although the SCL-90-OC subscale has been used in prospective OCS studies in the past [22] and has been adapted and widely applied in Greek adolescent, school and community samples [20, 70, 71], the use of more comprehensive, specific and sensitive OCS measurements should be adopted in future multilevel studies involving the school context.

Second, the use of a school sample to prospectively examine OCS raises questions considering the applicability of the findings to clinical samples. Therefore, similar longitudinal and contextualized studies are required to be replicated in clinical samples. Counterintuitively, the use of normative samples enables the dimensional investigation of OCS, which is significant for prevention purposes given their canonical distribution in the general population [4].

Finally, the sample was measured only twice and there was attrition between the two measurements. The 16–18 years old age range of participants coincides with a specific educational transition for Greek adolescents, which may narrow the applicability of the findings. Despite these factors, the study is novel in that there is a paucity of large cohort, longitudinal work in this area. Provided the statistical control of the attrition effects applied, the results contribute to the knowledge of school population differences, trait, and age-related factors affecting the experience of OCS. This study’s impact could be relevant both in an applied sense, for school interventions, and also in terms of widening further avenues of research relating to the school context and psychopathology. Future multilevel OCS studies within the school context should include samples of different cultural backgrounds, and investigate more than two time points and wider age ranges.

## Appendix

Level 1 equation:

$$Y = \varPi_{0} + \varPi_{1} *\left( {\text{time}} \right) + \varepsilon$$

Level 2 equations:

$$\varPi_{0} = \beta_{00} + \beta_{01} *\left( {\text{Emotional Stability}} \right) + \beta_{02} *\left( {\text{Gender}} \right) + \rho_{0}$$

$$\varPi_{1} = \beta_{10} + \beta_{11} *\left( {\text{Emotional Stability}} \right) + \beta_{12} *\left( {\text{Gender}} \right) + \rho_{01}$$

Level 3 equations:

$$\beta_{01} = \gamma_{010} + u_{01}$$

$$\beta_{01} = \gamma_{010} + u_{01}$$

$$\beta_{10} = \gamma_{100} + u_{10}$$

$$\beta_{11} = \gamma_{110} + u_{11}$$

*ε*, *ρ* and *u* parameters refer to controls of random effects at the three levels

*Note 1:* The data abides with the sample size requirements suggesting: (a) a minimum ratio of 10_clusters_/5_participants_ to test for fixed effects and cross-level interactions in models with one explanatory variable at each of the levels, and: (b) a minimum requirement of 30 clusters for testing standard errors of fixed effects [15, 16].

*Note 2:* Conducting covariance based structural equation modeling (CBSEM) was not selected as: (a) it requires at least three or four indicators (the current study includes two time points) for every latent variable (growth) [72] and; (b) it assumes multi-normal distribution of the observed variables to ensure meaningful results-which is rarely the case in empirical research [73]. Similarly, latent growth modeling (LGM) was not chosen as it assumes that Level 1 predictors with random effects have the same distribution across all participants in each subpopulation-while HLM allows different distributions [62]. Finally, HLM was preferred over partial least square analysis (PLS), as it estimates the effects of variables on the outcome variable at one level (i.e., individual), while at the same time taking into account the effect of variables on the outcome variable at another level (i.e., classroom) [62].
